# Landscape effects of a non-native grass facilitate source populations of a native generalist bug, *Stenotus rubrovittatus* , in a heterogeneous agricultural landscape

**DOI:** 10.1093/jis/14.1.110

**Published:** 2014-08-12

**Authors:** A. Yoshioka, M. B. Takada, I. Washitani

**Affiliations:** 1 Center for Environmental Biology and Ecosystem Studies, National Institute for Environmental Studies, 16-2 Onogawa, Tsukuba, Ibaraki 305-8506, Japan; 2 Graduate School of Agricultural and life Sciences, The University of Tokyo, 1-1-1 Yayoi, Bunkyo-ku, Tokyo 113-8657, Japan

**Keywords:** agroecosystem, alien plant, apparent competition, areawide pest management, generalist insect herbivore, metapopulation

## Abstract

Non-native plant species can provide native generalist insects, including pests, with novel food and habitats. It is hypothesized that local and landscape-level abundances of non-native plants can affect the population size of generalist insects, although generalists are assumed to be less sensitive to habitat connectivity than specialists. In a heterogeneous landscape in Japan, the relationship between the density of a native pest of rice ( *Stenotus rubrovittatus* (Matsumura) (Heteroptera: Miridae)) and the abundance of Italian ryegrass ( *Lolium multiflorum* Lam. (Poales: Poaceae)), a non-native meadow grass known to facilitate *S. rubrovittatus* , was analyzed. Statistical analyses of data on bug density, vegetation, and the spatial distribution of fallow fields and meadows dominated by Italian ryegrass, obtained by field surveys, demonstrated that local and landscape-level abundances of Italian ryegrass (the unmowed meadow areas within a few hundred meters of a sampling plot) positively affected bug density before its immigration into rice fields. Our findings suggest that a generalist herbivorous insect that prefers non-native plants responds to spatial availability and connectivity of plant species patches at the metapopulation level. Fragmentation by selective mowing that decreases the total area of source populations and increases the isolation among them would be an effective and environmentally-friendly pest management method.

## Introduction

Some native insect herbivores respond quite sensitively to invasions of non-native plant species in a wide range of ecosystems (Ellingson and Andersen 2002, Cronin and Haynes 2004 , de Groot et al. 2007 , Yoshioka et al. 2010a ). Such bottom-up effects on herbivore populations by non-native plants can cascade to upper trophic levels ( Burghardt et al. 2009 , Heleno et al. 2009 ) and seriously affect the entire food web in an ecosystem ( Tallamy 2004 ).

Most studies concerning bottom-up effects of plant invaders focused on negative local-scale effects on native insects through food limitation (e.g., Tallamy et al. 2010 ) and/or habitat modification (e.g., Yoshioka et al. 2010b ). Non-native plant invaders, however, can facilitate some generalist insect herbivores by providing trophic subsidies and/or novel habitats ( Rodriguez 2006 ), and thereby exert indirect negative effects (i.e., apparent competition *sensu*Holt 1977 ) on native plants and/or crops ( Malmstrom et al. 2005 , Carrière et al. 2006 ). Therefore, the facilitation of native generalist herbivores by non-native plants and the resulting dynamics may be an essential issue for predicting the effects of non-native plant invasions on biodiversity and ecosystem services.

The effect of the facilitation of native generalist herbivores by non-native plants is better considered at the landscape-level because stands of non-native plants are generally patchy and embedded in landscape elements such as croplands and woodlands. The spatial distribution of the patches can produce an “emergent” landscape-level effect of a plant invader on herbivore population dynamics, which differs from the effects of the summed area of the individual patches ( Bissonette 1997 , King 1997 ). For example, the metapopulation theory of Hanski (1999) holds that connectivity among habitat patches results in nonlinear positive effects on the population size of butterfly species ( Hanski and Ovaskainen 2000 ). To our knowledge, however, the positive landscape-level effects of non-native plants on native generalist insect herbivores have not yet been reported.

Agricultural landscapes in Japan are characterized by a fine-grained heterogeneous mosaic of cropland and various vegetated non-crop areas ( Washitani 2001 . Kadoya and Washitani 2011 ). This provides research opportunities for examining landscape-level effects of non-native plant species introductions and/or invasions on a generalist herbivore population. In Japan, the rice acreage reduction policy, practiced since 1970, has resulted in a prevailing trend in land-use change from paddies to meadows or fallow fields ( Hayashi and Nakazawa 1988 , Kiritani 2007 ). Italian ryegrass, *Lolium multiflorum* Lam. (Poales: Poaceae), a non-native meadow grass, has become a common forage crop in such meadows ( Hayashi and Nakazawa 1988 ).

*Lolium multiflorum* is the most important source host for a generalist mirid bug, the sorghum plant bug, *Stenotus rubrovittatus* (Matsumura) (Hemiptera: Miridae) ( Yoshioka et al. 2011 ), which is currently one of the most serious economic pests of rice *(Oryza sativa* L. (Poales: Poaceae) in Japan ( Kiritani 2007 ). *Stenotus rubrovittatus* preys on inflorescences of various species of Poaceae and Cyperaceae and has more than three generations per year ( Hayashi and Nakazawa 1988 , Kashin et al. 2009 ). Although *S. rubrovittatus* shows an opportunistic tactic (i.e., polyphagy and multivoltine brood strategy), the bug exhibits a marked preference to *L. multiflorum* inflorescences from early to midsummer, when the plant species produces seed heads ( Yoshioka et al. 2011 ). The mirid bug, however, only occurs in rice paddies during the short period when rice heads are present in midsummer ( Takeuchi et al. 2005 ). They reproduce little in rice paddies ( Takeuchi et al. 2005 ) but are assumed to spill over to rice fields from proximal meadows and fallow fields dominated by *L. multiflorum* . Therefore, meadows can be regarded as source habitats for the bugs, and the paddies are sinks. Fallow fields, which are sometimes invaded by non-native weeds including *L. multiflorum* , can also become source habitats for *S. rubrovittatus* .

Discoloration of rice grains attacked by the bugs causes severe economic loss to farmers under the current national regulation system for rice quality, which is based almost exclusively on the appearance of grains. Economically, the damage caused by the bugs is a serious problem, and effective pest management is urgently needed, especially in areas where biodiversity-friendly agriculture is practiced with no or reduced insecticide applications.

In this study, we aimed to determine whether the spatial characteristics of a heterogeneous agricultural landscape (landscape-level factor), especially the abundance and/or connectivity of meadow patches dominated by non-native *L. multiflorum* , as well as the local abundance of a host plant (local-level factor), affect the density of *S. rubrovittatus* in their source habitats during the critical stage before immigration into paddies.

The spatial distributions of meadows dominated by *L. multiflorum* , fallow fields, and woodlands as landscape-level factors were surveyed in an area of Osaki City, Miyagi Prefecture, northern Japan, where biodiversity-friendly farming with no or fewer agrochemicals was practiced. Densities of mirid bug populations in fallow fields and meadows were measured in early August, with the abundances of potential hosts (Poaceae and Cyperaceae) as local-level factors. Then, the relationships between *S. rubrovittatus* density and local/landscape-level factors were analyzed with spatial statistics.

## Materials and Methods

### Study area

The study was conducted in the Tajiri area of Osaki City, Miyagi Prefecture, northern Japan (38º37′N, 141º07′′E), where pioneering biodiversity-friendly (no or reduced application of pesticide and fertilizer) agriculture has been practiced. The landscape of the area mainly consists of paddies, fallow fields, meadows converted from paddies within the past few decades, and woodlands. Annual precipitation in the city in 2008 was 1,126 mm; mean temperatures in June, July, and August were 18.5ºC, 22.6ºC, and 22.5ºC, respectively (data from the Japanese Meteorology Agency, http://www.jma.go.jp/jma/index.html , accessed 9 April 2009).

*Lolium multiflorum* , which has been cultivated as a common forage crop and has naturalized to become an invasive alien plant ( Miyawaki and Washitani 2004 ), was the most common meadow grass cultivated in meadows in the study area. The agricultural field area of Italian ryegrass meadows ranged from ≈60 to 20,000 m ^2^ in the study area (note: an “agricultural field” in this paper is defined as a spatially–continuous agricultural unit where crop management was homogenously conducted). *Stenotus rubrovittatus* is the most abundant and influential arthropod pest in the paddies of the area ( Kashin et al. 2009 , Yoshioka et al. 2011).

In meadows of the study area, *L. multiflorum* is sown in autumn or early spring and harvested for forage in the middle of June, but grass mowed in the early summer rapidly regrows and is harvested again in late July. The timing of the second harvest is highly variable according to summer weather conditions because farmers prefer drying mowed grass in the sun before collecting it from the meadow (A. Yoshioka, personal observation). Thus, in years with a rainy summer, some meadows remain unmowed until early- to mid-August, when rice plants begin to head and *S. rubrovittatus* seasonally colonizes paddy fields. In autumn, most of the meadows and fallow fields are dominated by gramineous weeds such as *Echinochloa* spp. after most *L. multiflorum* has become senescent.

In fallow fields, sedges and grasses such as *Carex eurocarpa* Maxim. (Poales: Cyperaceae), *Schoenoplectus juncoides* (Roxb.) Palla (Cyperaceae), *Agrostis gigantea* Roth (Poaceae), *Echinochloa* spp. (Poaceae), and a few naturalized *L. multiflorum* come to head in early August.

### Landscape structure

Field surveys were conducted to assess the spatial distribution of landscape elements and boundaries of meadow and fallow fields in the study area. The surveys were conducted from mid-June to early July and from the end of July to mid-August in 2008. To conduct the landscape survey efficiently, a map of agricultural land use and information compiled by the local government on crop rotation were used for mapping spatial distribution of each landscape elements roughly in advance.

**Figure 1.**
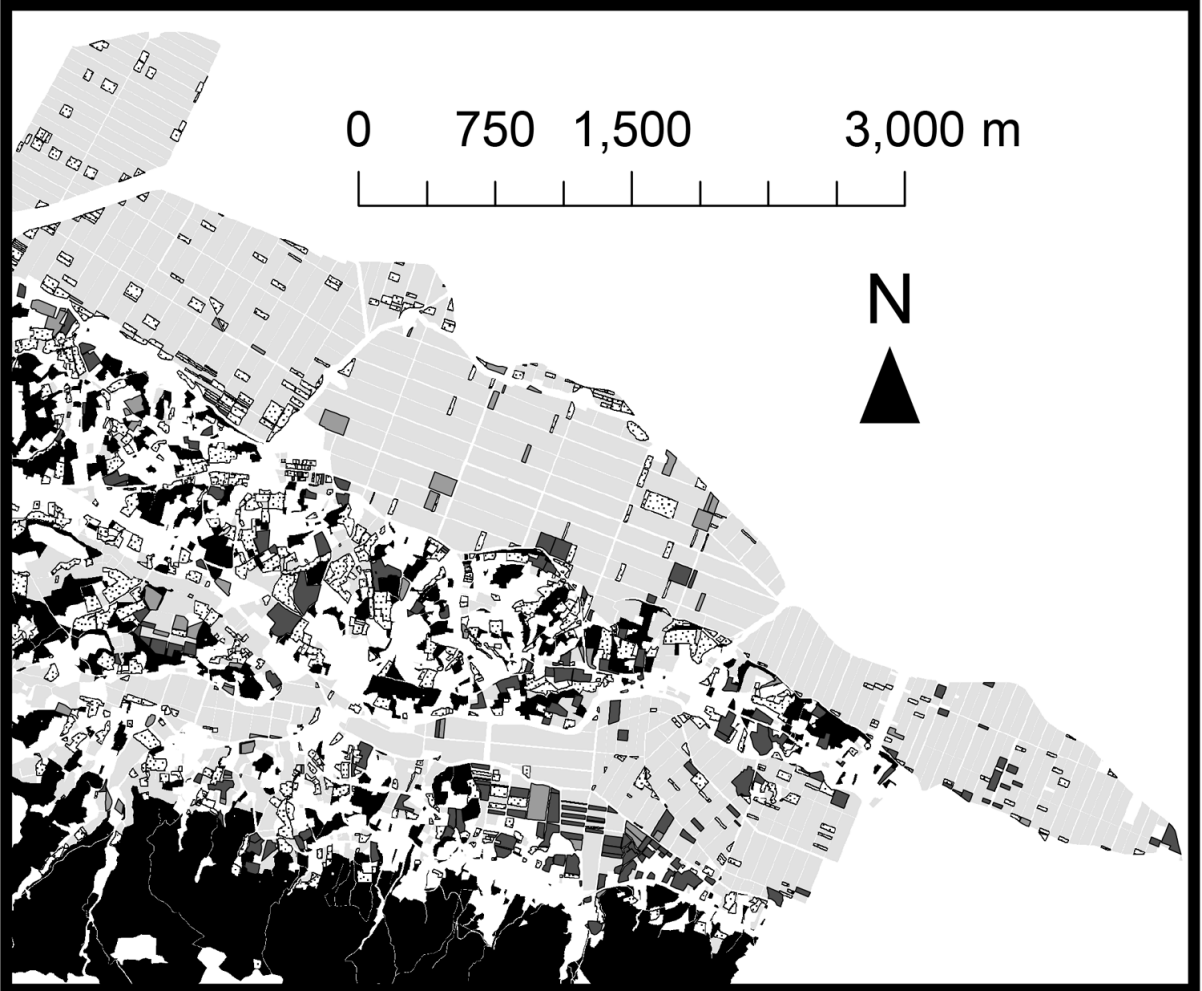
The land-use map of study area (Tajiri area of Osaki city, Miyagi Prefecture) in summer of 2008, based on information of the local government and filed survey. Dark grey and grey polygons indicate mowed and unmowed meadows, respectively. Dotted white polygons indicate fallow fields. Light grey and black polygons indicate paddies and woodlands, respectively. Note that boundary of paddy fields were omitted. High quality figures are available online.

Five categories of land-use were identified and mapped ( Fig. 1 ): paddy fields, Italian ryegrass meadows, fallow fields, woodlands, and others. Although the local government compiled reports from farmers about locality of fields where Italian ryegrass was cultivated, a part of the fields was inhabited by no or little Italian ryegrass when the authors conducted their survey. In such cases, Italian ryegrass meadows were defined as agricultural fields where the percent cover of heading *L. multiflorum* was >10% from late June to early August (including meadows mowed in late July). The meadows were subdivided into two categories, “mowed” and “unmowed,” according to whether or not they had been mowed in late July. Among these landscape elements, surrounding woodlands were assumed to be a source of natural enemies ( Amano et al. 2011 ) and/or dispersion barriers for the grassland bug, and unmowed meadows and fallow fields were assumed to function as suitable habitat and dispersal routes for the bugs. The other landscape elements, except mowed meadows (see below), which were assumed to be a matrix or sink for the bug metapopulation, were not analyzed here, in order to avoid multicol-linearity. The data collected were mapped and analyzed using the software packages ArcGIS 9 (ESRI, Redlands, CA) and Hawth’s Analysis Tools ( http://www.spatialecology.com ).

### Field sampling

The field censuses of *S. rubrovittatus* and its host plants were conducted in the meadows and fallow fields from 1 to 9 August 2008, at the peak density of the bug population in the first annual generation before seasonal dispersion into paddies ( Kashin et al. 2009 ). In total, 194 sampling plots (37 in unmowed meadows, 43 in mowed meadows, and 114 in fallow fields) were censused within about 20-km ^2^ area that included sites dominated by various Poaceae and Cyperaceae species. Note that a census “plot” in this study was defined as a representative point in an agricultural field. Each census plot was located near the center of a meadow or fallow field. The distance to the nearest neighbouring plot was 102 ± 79 m (mean ± SD). The average distances to the nearest neighboring plot among unmowed meadows, mowed meadows, and fallow plots were 260 ± 230 m, 131 ± 104 m, and 123 ± 86 m (mean ± SD), respectively.

Daily censuses started at 09:00 and ended at 17:00. In each plot, the density of adult *S. rubrovittatus* was measured once by sweep-net sampling (20 sweeps using a 36-cm diameter insect net).

The abundance of inflorescences of each Poaceae and Cyperaceae species was also measured by counting the number of inflorescences within a 0.2 × 6-m ^2^ quadrat set near the plot. Vegetation height also was recorded at nine regularly spaced points within each quadrat and averaged over each plot. Whether or not a meadow plot had been mowed recently also was recorded. Further details of the sampling methods are described in Yoshioka et al. (2011) , in which the data set including the data used in this study was analyzed to reveal host preferences of *S. rubrovittatus* .

### Statistical analyses

To analyze the effects of local and landscape elements on *S. rubrovittatus* , a generalized linear model with a negative binomial error distribution was applied. The data from un-mowed meadows and fallow fields were analyzed separately because bug density is thought to be affected differently by factors between these habitats ( Yoshioka et al. 2011 ). The data from mowed meadows without *L. multiflorum* heads at the time of sampling were not included in the analysis.

The models included bug density in focal plots of 37 unmowed meadows or 114 fallow fields as a response variable and landscape factors (unmowed meadow, fallow field, and woodland area surrounding the focal plots), and within-plot factors (vegetation height and inflorescence abundance of each Poaceae and Cyperaceae species that was recorded in >10 plots in each habitat type: *L. multiflorum* in mowed meadow plots and *C. eurocarpa, Schoenoplectus juncoides* (Roxb.) Palla, *A. gigantea, Echinochloa* spp., and *L. multiflorum* in fallow plots) as independent variables. The latitude and longitude of each study plot also were included in the models as independent variables to separate the effects of unexpected geographical trends ( Legendre and Legendre 1998 ).

Takada et al. (2012) and Yasuda et al. (2011) suggested that the density of *S. rubrovittatus* in paddy fields was significantly affected by landscape elements within 300-400 m of focal sampling plots. To explore the effective spatial scale in this study, increasing radii of 100, 200, 300, and 400 m were established around each sampling plot, and the proportion each radius represented by the three landscape elements was calculated using ArcGIS. In addition, the area of an agricultural field where a focal plot existed, rather than patch connectivity represented by the summed area of landscape elements at various radii from the focal plot ( Moilanen and Nieminen 2002 ), may play an important role. To address such cases, a model using the area of a field where the focal plot exists rather than surrounding area at various radii from the focal plot was also analyzed. Unlike natural ecosystem, as defined in Hanski (1999) , field area (corresponding to patch area) in this study system was determined by the size of crop management unit by a farmer rather than the quality of habitat.

Model selection was performed using Akaike's information criterion (AIC) to compare candidate models consisting of all possible combinations of explanatory variables. The AIC was tested for each radius size to obtain the best model (i.e., the model with the lowest AIC). The values of within-plot factors remained unchanged with changing radius size. Although model selection with “all possible models” runs the risk of selecting an inappropriate model as a result of data-dredging unless the candidate models are biologically meaningful ( Burnham and Anderson 2002 ), our previous studies ( Yoshioka et al. 2011 ) confirmed that these explanatory variables are sufficiently biologically meaningful. Before analysis, all of the explanatory variables were centered and scaled (divided by the SD) so that their effects could be compared.

Spatial structure of Italian ryegrass meadows in the study area (i.e., mowed + unmowed meadows) is expected to serve as a longer-term index of connectivity among source habitats of *S. rubrovittatus.* To clarify how short-term landscape-level changes in meadow status (i.e., unmowed meadow in early August) rather than long-term landscape structure affect bug density, a similar analysis with surrounding all Italian ryegrass meadows (including unmowed and mowed areas) instead of only unmowed meadows was also conducted. If the best-fit models with surrounding unmowed meadow areas showed better fits (lower AIC values) than those with surrounding all Italian meadow areas, the presence of a short-term landscape effect would be supported.

All statistical analyses were performed using R for Windows 2.15.1 ( R Development Core Team 2012 ). For the best models, multicollin-earity among independent variables was checked using tolerances. Spatial autocorrelation of residuals in the best models was also tested by Moran’s I correlograms ( Legendre and Legendre 1998 ), which plot Moran’s I in 20 100-m-wide distance classes. Moran’s I and its *P* -value, based on 10,000 permutations, were computed using the *ncf* package in R, and the significance level was corrected using the Bonferroni method as recommended by Legendre and Legendre (1998) . In addition, these GLMs incorporating a random effect (i.e., Generalized linear mixed model; GLMM, see Bolker et al. [2009] for details) also were reconstructed to confirm that overlap of radii did not violate independence of sampling plots. At each radius size, if radii from plots overlapped each other, the plots were treated as the same group. Then, GLM with the same variables in addition to random effect by the group were constructed by *glmmADMB* package in R.

## Results

### Choice of spatial scale for models with good performance

**Fig 2.**
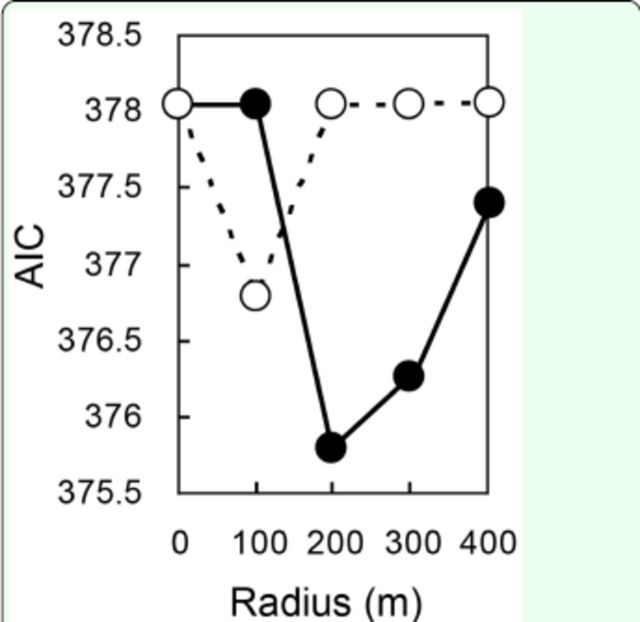
The minimum AIC values of the models explaining density of *Stenotus rubrovittatus* in unmowed plots for each radius size. The values of models including unmowed meadow area as one of the candidate independent variables are shown by filled circles, while those of models including all Italian ryegrass meadow area (mowed and unmowed) are shown by open circles. High quality figures are available online.

**Figure 3.**
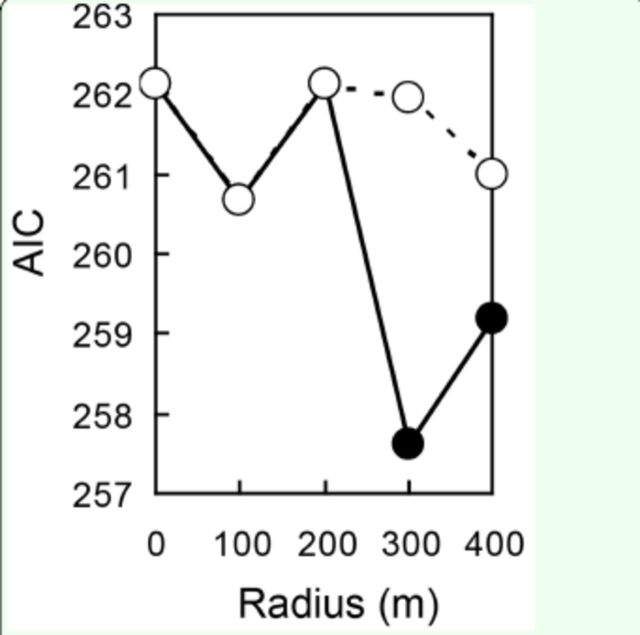
The minimum AIC values of the models explaining density of *Stenotus rubrovittatus* in fallow plots for each radius size. The values of models including unmowed meadow area are shown by filled circles, while those of models including all Italian ryegrass meadow area (mowed and unmowed) are shown by open circles. High quality figures are available online.

Model selection revealed that landscape elements within a radius of 200 m from the focal plots of unmowed meadows minimized AIC values in models of bug density in unmowed meadow plots ( Fig. 2 ), whereas those within a radius of 300 m from the focal plot minimized AIC values in models of bug density in fallow plots ( Fig. 3 ). These AIC values were considerably lower (AAIC > 2) than the most parsimonious models with no landscape factors included, i.e., 0-m-radius models (AIC = 378.05 and 262.13 in unmowed meadow and fallow plots, respectively). The tolerances of each selected variable were sufficiently high (> 0.76) in both of the most parsimonious models. No significant Moran’s I values were detected from the residuals of the best models. GLMMs incorporating overlaps of radii also revealed that random effects were quite small to their standard error and did not affect the direction and significance of the coefficients and rank of AIC at each radius. Thus, assumption of independence of bug density among plots was considered reasonable.

### Factors affecting bug density in unmowed meadow plots

In the most parsimonious model for unmowed meadow plots with landscape elements within a radius of 200 m, density was highly correlated with unmowed meadow area around the plot and the local abundance of *L. multiflorum* inflorescences ( Table 1 ). The direction of the coefficients of the two factors was consistently maintained in the other candidate models with landscape elements within a 200-m-radius, with ∆AIC <2 ( Table 1 ). The best model with landscape elements within a radius of 300 m also showed that the two factors positively affected bug density.

**Table 1.**
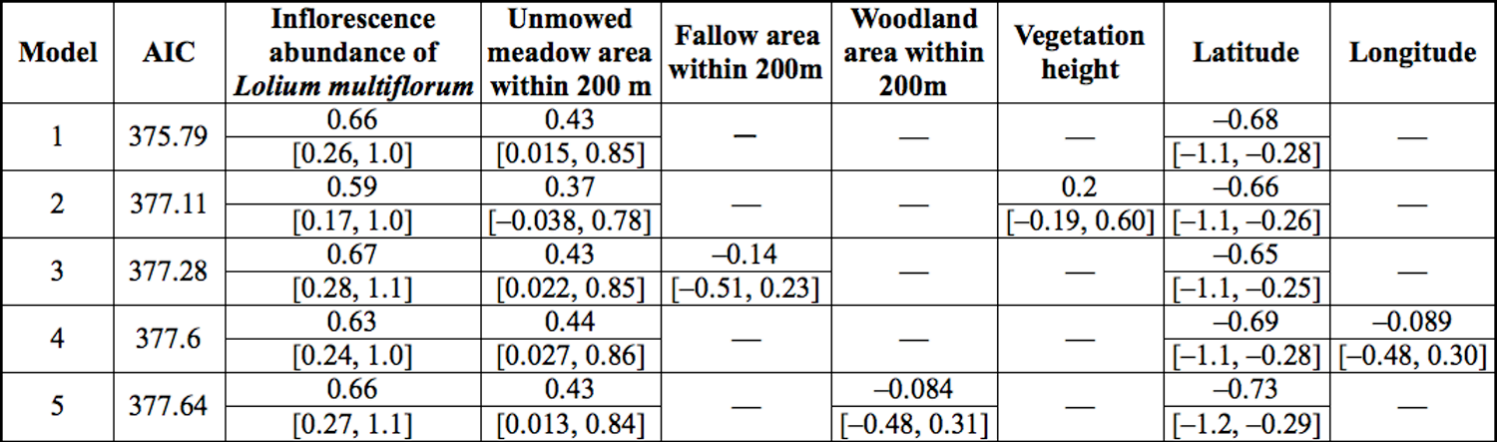
The best five generalized linear models explaining the density of adult *Stenotus rubrovittatus* in meadows in the model selection at 200 m radius and their coefficients [95% C.I.a].

^a^ 95% Wald Confidential Intervals

The model including field area of the focal plot of unmowed meadow instead of the summed unmowed meadow area within a radius of 200 m from the plot had a higher AIC value (379.97), and the 95% CI of the coefficients of field area overlapped zero (-0.30, 0.53). Thus, the effect on the bug may be less related to the size of the focal unmowed meadow field than to the connectivity of surrounding unmowed meadows.

### Factors affecting bug density in fallow plots

Mirid bug density in fallow plots was positively correlated with unmowed meadow area within a radius of 300 m ( Table 2 ), but not with surrounding fallow area. The direction of the coefficients of the factors was maintained in the other candidate models with landscape elements within a radius of 300 m and **A** AIC < 2. Some within-plot factors (abundances of *S. juncoides, A. gigantea, Echinochloa* spp., and *L. multiflorum* inflorescences) also had positive effects on density. Surrounding unmowed meadow areas within a radius of 400 m also were positively correlated (lower 95% CI of the coefficient > 0) with bug density.

**Table 2.**
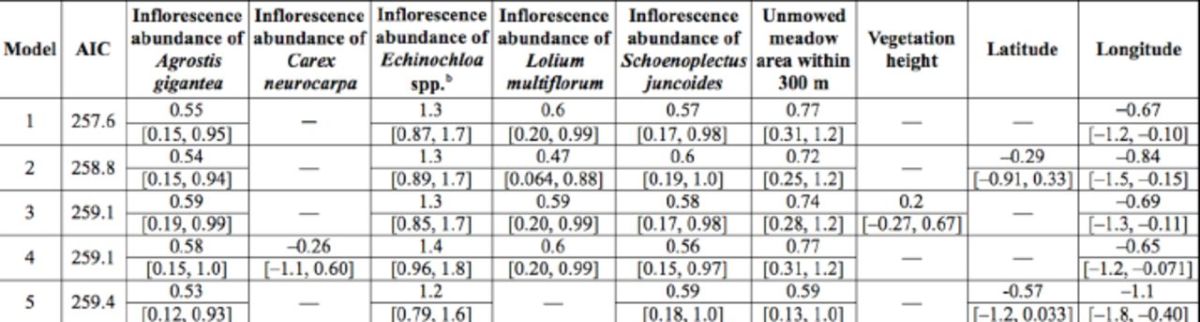
The best five generalized linear models explaining the density of adult *Stenotus rubrovittatus* in fallow fields in the model selection at 300 m radius and their coefficients [95% C.I.a].

^a^ 95% Wald Confidential Intervals

^b^ *Echinochloa crus-galli* (L.) P. Beauv. (Poaceae) and *Echinochloa oryzicola* (Vasing.) Vasing. (Poaceae)

### Models with all italian ryegrass meadow area (mowed and unmowed)

The models for bug density in unmowed meadow or fallow plots with the total area of surrounding all Italian ryegrass meadows (mowed + unmowed) generally showed a poorer fit than models with only the total surrounding unmowed meadow area ( Fig. 2 , 3). Among the models for bug density in unmowed meadow plots that included the total area of surrounding Italian ryegrass meadows as an independent variable, no landscape factors were selected in the best model ( Fig. 2 ). The exceptions were the models with landscape elements within a radius of 100 m; these models showed a positive effect of summed Italian ryegrass meadow area, which was moderately correlated with the summed unmowed meadow area within a radius of 200 m ( *r* = 0.70).

The most parsimonious model for the bug density of fallow fields with summed surrounding unmowed meadow area as an independent variable had a considerably lower AIC value (AAIC < 2) than the most parsimonious model with the total area of all Italian ryegrass meadows ( Fig. 3 ).

## Discussion

### Landscape effects of a non-native grass on the density of a native mirid bug

This study demonstrated that local (i.e., abundance of *L. multiflorum* inflorescences) and landscape (i.e., surrounding unmowed meadow area within a few hundred meters) factors had significant positive effects on *S. rubrovittatus* density in unmowed meadows and fallow sites scattered in an agricultural landscape. Thus, *L. multiflorum,* a non-native plant species, significantly influenced the density of a generalist bug at local and landscape levels. This positive multilevel effect of unmowed meadow fields on bug density suggests that increases in surrounding unmowed meadow area would cause sharp increases in bug abundance (i.e., density × area) in a focal field and, therefore, within the entire metapopulation.

Our result that unmowed meadow areas (rather than all meadows) appropriately explain the observed pattern also suggests that habitat fragmentation can affect the dynamics of generalist insect populations in a relatively short term (within a generation), probably through decreases in habitat area and degree of isolation from other suitable habitat patches. This is similar to results previously shown for harvest mice inhabiting fallow patches ( Kuroe et al. 2010 ) and a planthopper species seasonally specialized on oats ( Grilli and Bruno 2007 ). Although the model for bug density in un-mowed meadows with the total area of all Italian ryegrass meadows within a radius of 100 m also had a relatively low AIC value, it was inconsistent with models at other spatial scales and with the results of previous studies ( Carrière et al. 2006 , Yasuda et al. 2011 , Takada et al. 2012 ). In addition, the sum of the area of Italian ryegrass meadows over mowed and unmowed areas within a radius of 100 m was moderately correlated with the sum of unmowed meadow areas within a radius of 200 m; thus, the correlation with bug density may be spurious. The local summer density of the bugs in a habitat patch may be relatively independent of longer time scale dynamics of metapopulations, as discussed below.

### Possible mechanisms for the short-term landscape-level effect

Positive short-term effects of habitat area and/or connectivity on insect density similar to the one revealed in this study have been reported repeatedly ( Root 1973 , Andow 1991 , Cronin and Haynes 2004 , Matter et al. 2004 , Grilli and Bruno 2007 , Haynes and Crist, 2009 ). Generally, such temporal effects can be explained by the resource concentration hypothesis ( Root 1973 ) with the assumption of dispersal-driven mortality ( Cronin and Haynes 2004 , Matter et al. 2004 ). That is, phytophagous insects tend to emigrate from smaller habitat patches at relatively small spatial scales ( Haynes and Crist 2009 ), while increasing time spent in unsuitable habitats (i.e., “matrix” in a metapopulation; Hanski 1999 ) drives higher mortality in a more fragmented landscape with spatially separated suitable habitat patches ( Hanski et al. 2000 ). In our study area, spiders ( Kobayashi et al. 2011 , Takada et al. 2013 ) and frogs (Iwabuchi, personal communication) in paddy fields (i.e., the matrix for *S. rubrovittatus* in the first generation) were observed preying on *S. rubrovittatus* in summer. These predators may contribute to dispersal-driven mortality of the bug over a short time scale. Interactions among natural enemies, however, should be carefully studied for evaluation of pest control at the complex agricultural landscapes ( Martin et al. 2013 ).

If dispersal-driven mortality is the major factor determining the emergent pattern observed in this study, even generalist herbivores may be subjected to immediate regulation by the spatial availability and connectivity of habitats, like specialist insect herbivores, although generalists have been assumed to be fairly resistant to habitat fragmentation ( Ockinger et al. 2010 ). This may be true for many generalist insect herbivores because most of them are known to show marked preferences to one or a few particular host plants ( Fox and Morrow 1981 , Lu et al. 2010 ). In addition, our results suggest that these positive emergent effects can arise at finer landscape scales (within a few hectares), and that they can be caused by the establishment and dominance of a non-native plant even in a small part of a landscape.

### Implications for pest management in invaded agricultural landscapes

Some implications for pest management can be drawn from the current results. Several studies on apparent competition (e.g., Carrière et al. 2006 , Orrock et al. 2008 ) have reported a “spillover of pests” from source populations, fostered by non-native plant patches, to sink or ephemeral populations, affecting native plants or crops. An understanding of dispersal-related mortality and its tendency to be affected by the landscape should be essential to integrated pest management.

Source populations, rather than sink populations, should be managed to control the metapopulation as a whole. If the habitat area and/or connectivity among source patches is an important factor affecting the source populations, as was partly shown in the present study, removal of some non-native host plant patches, by decreasing the total area and increasing the isolation of the patches, is expected to be effective at decreasing metapopulation size (or persistence). Therefore, when designing landscape-oriented pest control, recognition of source-source connectivity, as well as source-sink connectivity, should be important, as has been suggested in previous studies ( Carrière et al. 2006 , Orrock et al. 2008 ).

The landscape-level effects revealed in this study indicate that a spatially realistic metapopulation approach is not only needed for the conservation of particular endangered species ( Harrison 1991 ), but it is likely to be effective for pest management without insecticides in agricultural landscapes with high habitat heterogeneity (e.g., Washitani 2001 , Benton et al. 2003 ).
